# Current status of transcatheter intervention for complex right ventricular outflow tract abnormalities

**DOI:** 10.21542/gcsp.2024.7

**Published:** 2024-01-03

**Authors:** Yoshiyuki Kagiyama, Damien Kenny, Ziyad M. Hijazi

**Affiliations:** 1 Department of Pediatric Cardiology, Children’s Health Ireland at Crumlin, Dublin 12, Republic of Ireland; 2 Department of Pediatrics and Child Health, Kurume University School of Medicine, Kurume, Japan; 3 Department of Cardiovascular Diseases, Sidra Medicine, and Weill Cornell Medical College, Doha, Qatar

## Abstract

Various transcatheter interventions for the right ventricular outflow tract (RVOT) have been introduced and developed in recent decades. Transcatheter pulmonary valve perforation was first introduced in the 1990s. Radiofrequency wire perforation has been the approach of choice for membranous pulmonary atresia in newborns, with high success rates, although complication rates remain relatively common. Stenting of the RVOT is a novel palliative treatment that may improve hemodynamics in neonatal patients with reduced pulmonary blood flow and RVOT obstruction. Whether this option is superior to other surgical palliative strategies or early primary repair of tetralogy of Fallot remains unclear. Transcatheter pulmonary valve replacement has been one of the biggest innovations in the last two decades. With the success of the Melody and SAPIEN valves, this technique has evolved into the gold standard therapy for RVOT abnormalities with excellent procedural safety and efficacy. Challenges remain in managing the wide heterogeneity of postoperative lesions seen in RVOT, and various technical modifications, such as pre-stenting, valve ring modification, or development of self-expanding systems, have been made. Recent large studies have revealed outcomes comparable to those of surgery, with less morbidity. Further experience and multicenter studies and registries to compare the outcomes of various strategies are necessary, with the ultimate goal of a single-step, minimally invasive approach offering the best longer-term anatomical and physiological results.

## Introduction

Approximately 20% of patients with congenital heart disease (CHD) have multi-level right ventricular outflow tract (RVOT) abnormalities^1^ and the incidence of these specific types of CHD has been growing over the past three decades^2^. Until recently, only surgical repair or palliative therapy was available for complex RVOT abnormal lesions, such as pulmonary valve atresia, severe RVOT obstruction with tetralogy of Fallot, or postoperative pulmonary regurgitation or obstruction. However, with the evolution of radiofrequency perforation of the pulmonary valve, right ventricular outflow tract stenting, and transcatheter valve implantation, these patients can now receive transcatheter intervention as a less invasive and more effective alternative therapy. In this review, we discuss the current status of three representative transcatheter interventions for complex RVOT abnormalities.

## Transcatheter pulmonary valve perforation

### Background

Transcatheter perforation of the atretic membranous pulmonary valve was introduced in the 1990s as an alternative to surgical RVOT reconstruction for patients with pulmonary atresia and intact ventricular septum (PAIVS)^1,3–6^. Pulmonary circulation is dependent on the patent ductus arteriosus (PDA) in PAIVS patients, and the initial step of treatment for such patients is the establishment of adequate pulmonary blood flow (PBF). Perforation of the pulmonary valve is one of these options by creating RV to pulmonary artery (PA) continuity, with the ultimate goal of biventricular repair. Although hemodynamically favorable biventricular repair is the goal, it is difficult to achieve in patients with hypoplastic RV or coronary artery abnormalities. In the early days, because of the rarity of the condition and the morphological heterogeneity of PAIVS, the treatment strategy was not standardized and profoundly different between institutions^7^. With growing recognition about the risks of “aggressive” biventricular repair, currently approximately 40–60% of PAIVS patients receive this procedure to pursue biventricular repair^7–9^.

### Preprocedural evaluation

The initial treatment strategy is selected based on factors such as the presence and extent of the ventriculocoronary connections, morphology of the RV and tricuspid valve (TV), and type of pulmonary atresia.

The existence and extent of the ventriculocoronary connections should be clarified before pulmonary valve perforation. Suprasystemic RV pressure from fetal life acts to maintain embryological ventriculocoronary connections, that is, sinusoidal communications with collision of conflicting blood flow from the aorta and RV inside the coronary artery (CA) cause potential distortion and stenosis of the coronary sinusoids^1^. In the presence of right ventricular dependent coronary artery circulation (RVDCC), ventriculocoronary connections with stenotic or obstructive antegrade CAs, reduction of blood flow or pressure in the RV may cause myocardial ischemia and death^10^. Therefore, patients with a broad area of myocardium supplied by the RVDCC are recognized as contraindications for valve perforation and subsequent RV decompression and are considered candidates for univentricular repair or heart transplantation^11–13^.

**Figure 1.**
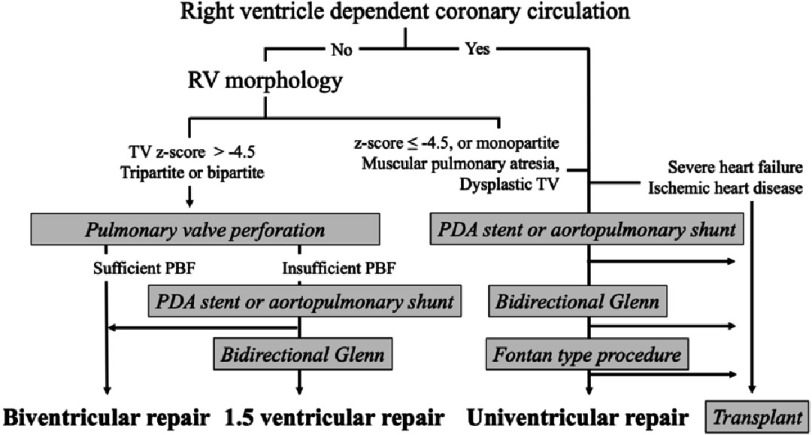
Flowchart of current treatment strategy for PAIVS. Abbreviations: PBF, pulmonary blood flow; PDA, patent ductus arteriosus; RV, right ventricle; RVD, right ventricular decompression; TV, tricuspid valve.

It is important to objectively evaluate RV size, diameter, and z-value of the TV on echocardiography^5,14^. The ratio of the TV to the mitral valve, RV inlet length z-score, and diastolic RV area z-score were also useful^15^. According to these studies, a TV z-score of over −2.5 SD is recognized as a good marker for biventricular repair and with TV z-score of −4.5 to −2.5 SD considered a borderline RV. In patients with borderline RV size, a sufficient increase in PBF immediately after successful valve perforation is not guaranteed, and some patients require an additional PBF source, such as a surgical shunt or stent into the PDA or RVOT^8,16^. However, it is well known that RV growth is favorable after pulmonary valve perforation and RV decompression^17–19^. Even if RV growth is insufficient to support full cardiac output due to the limited size or reduced compliance^20^, some patients procced to achieve a one-and-a-half ventricular repair by placing a bidirectional Glenn anastomosis. Therefore, in patients without RVDCC, an attempt at initial valve perforation may be worthwhile, even with a borderline RV^8^. Muscular-type pulmonary atresia with poorly developed infundibulum or severely dysplastic or Ebstein-like TV is a morphological contraindication for transcatheter perforation. Figure 1 shows the flowchart of the current treatment strategy for PAIVS.

### Valve perforation and balloon dilation

**Figure 2.**
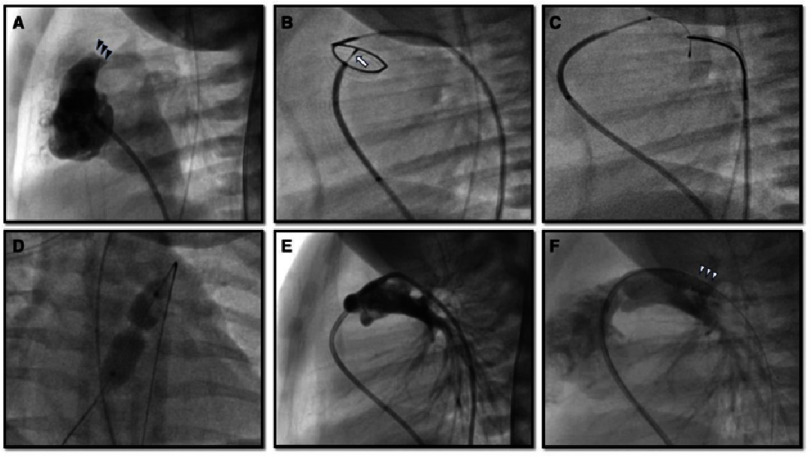
Pulmonary valve perforation. (A) Preprocedural right ventricular outflow angiography shows an atretic pulmonary valve (black arrowheads) without antegrade flow. In frame B, a radiofrequency wire (arrow) perforated and passed the atretic pulmonary valve within a loop snare which was advanced retrogradely via the patent ductus arteriosus. The wire was snared and pulled into the descending aorta to establish a wire loop (C). An angioplasty balloon was inflated over the wire at the pulmonary valve (D). (E) Post Pulmonary angiography status post pulmonary valve perforation (E) and (F) post placement of the patent ductus arteriosus stent (white arrowheads).

This procedure (Figure 2) is usually performed under general anesthesia, with ductal patency maintained with prostaglandin E_1_ infusion. Prior to pulmonary valvular perforation, the nature of pulmonary atresia and RV hypoplasia, size of the pulmonary valve plate, infundibulum, RV, and PDA were evaluated using angiography. RVDCC should be excluded with RV angiography and/or aortography^15^. A 4 or 5 Fr Judkins right coronary catheter is maneuvered into the infundibulum *via* an antegrade approach. Location of the atretic pulmonary valve is confirmed with intermittent contrast injection and the catheter is placed to enface the center of the valve. Placing another angiographic or snare catheter into the main pulmonary artery across the PDA *via* a retrograde approach enables coaxial central alignment of the catheters.

Initially, transcatheter valve perforation equipment included a laser wire, the stiff end of a coronary wire, and radiofrequency (RF) wire. Currently, RF wire, which is designed to create a controlled hole in soft tissue by delivering radiofrequency (RF) energy, is widely utilized, with a high success rate of 91–94% and low complication rates^21,22^. A Nykanen RF wire (Baylis Medical, Montreal, Canada) is the most commonly used wire with 0.016” rounded tip and 0.024” wire covered with Teflon insulation^22^. To perforate the atretic valve, the traditional application of 3-10 Watts/sec for 2-5 s RF energy has been recommended^23^. However, a recent multicenter retrospective study reported that higher RF energy increased the risk of cardiac perforation^24^, therefore minimal optimal application of RF energy may be necessary. The high cost and low availability of RF generators and wires are other important considerations, particularly in developing countries.

With the refinement of chronic total occlusion (CTO) hardware technology, mechanical valve perforation using a CTO wire has been introduced as a technique with lower cost and risk of complications^25–28^. Because CTO wires are specially designed to penetrate occluded coronary arteries, they can provide better penetrability, straightforward pushability, and stability during perforation, which can reduce catheter displacement and subsequent cardiac perforation^16^. On the contrary, success rate in previous reports was as high as RF perforation^26,27,29^. The use of coronary microcatheters has been reported to enhance stabilization and increase success rates^30^.

Once the RVOT to PA continuity is established and confirmed with angiography, a microcatheter is advanced into the main PA and the RF wire is replaced by a 0.014” coronary wire. Thereafter, over this wire in the distal PA or the descending aorta through the PDA, serial balloon dilation of the pulmonary membrane to up to 140% of the valve annulus size or 7-8 mm diameter will be performed to achieve adequate PBF and RV decompression. To enhance trackability of the balloon system over the wire, many operators prefer to snare the wire in the descending aorta^31^.

### Additional procedure or re-intervention

If the patient remains deeply cyanotic (saturation less than 80%), PDA stenting or surgical aorto-pulmonary shunt creation may be performed to augment the PBF. This could be performed in the same setting or several weeks after the initial valve perforation. Generally, the RV remains restrictive for a few weeks and it is difficult to predict which patients will require an additional source of PBF. Previous reports suggest that almost half of the patients required additional procedures to augment PBF after initial right ventricular decompression. Risk factors associated with failure to achieve a biventricular circulation include: small RV area, tricuspid valve hypoplasia, small RV to LV size ratio, and mild or lesser degrees of tricuspid valve regurgitation (Table 1)^17,18,32,33^.

**Table 1:** Recent studies which advocated pre-procedural predictors of final circulatory status.

**Authors, year**	**Petit 2017**	**Chen 2018**	Maskatia 2018 ^*^	Yoldaş 2020
**Patients number (group)** **Group assignment**	n 99	n 36, (26 / 5) BVR / not BVR	n 81	n 31, (16 / 15) RVD success /not
**Tripartite : Bipartite**	not shown	83% : 17%	not shown	65% : 35%
**TV annulus diameter, mm**	median 10.5	not shown	not shown	not shown
**TV z-score**	median −0.86	mean −3.34 vs −6.86^a^	median -0.64	mean -0.64 vs -1.18
**RV area, cm** ^**2**^ ** or area index**	median 2.0	not shown	median 12.1 (/m^2^)	mean 10.7 vs 8.8 (/m^2^)
**TV/MV ratio**	median 0.85	median 0.80	not shown	mean 0.84 vs 0.92
**TR ≥ moderate**	69%	not shown	61%	not shown
**Procedural success rate**	94%	92%	94%	48%
**In-hospital mortality**	0%	7% (3.5% by sepsis)	0%	19% (all by sepsis)
**Require re-dilatation**	64%	not shown	not shown	54%
**Require additional PBF**	45%	28%	46%	52%
**Achievement of BVR**	83%	84%	86%	48%
**Factors associated with BVR achievement**	not advocated	TV/MV ratio > 0.79	RV area index > 6 cm^2^/m^2^	TV/MV > 0.85 TV z-score > -1
**Factors associated with failure to achieve BVR**	≤mild TR^b^, lower RV area	Bipartite / history of BAS	not advocated	not advocated

**Notes.**

Abbreviations BVR biventricular repair PBF pulmonary blood flow RV right ventricle RVD right ventricular decompression RVOT right ventricular outflow tract TR tricuspid regurgitation TV tricuspid valve

* post hoc analysis data of Petit’s.

a < − 2.5 SD 19%, −5 SD 50%.

b not significant in its post hoc analysis.

### Mortality and morbidity

Only a few studies have summarized the complication rates after this procedure. Perforation of the RVOT or PA is the most hazardous complication with prevalence of 10–19%^6,24,34^. Petit et al. reported that this complication was associated with the use of an RF wire and a greater degree of RF energy^24^. They also mentioned that arrhythmias were the most common complications, occurring in 61% of patients and requiring intervention in their cohort. Postprocedural major adverse cardiac events such as death, stroke, cardiopulmonary resuscitation, or mechanical circulatory support occurred in approximately 20% of patients in a recent multicenter study; nonetheless, this percentage was lower than that after single ventricular palliation, even when RVDCC patients were excluded from the analysis^9^.

### Summary

Wire perforation to the atretic pulmonary valve is an established therapy with excellent results. However, indication remains controversial because of the morphological and physiological diversity of PAIVS. Both RF and CTO wires are effective; however, further development of knowledge and experience with CTO wires is necessary. Ultimately, it may be possible to provide transcatheter pulmonary valve replacement in these patients later in life, thereby providing complete percutaneous repair for this condition in some patients. Additionally, this technique may also be performed as palliative treatment for pulmonary atresia with ventricular septal defects in combination with RVOT stenting, particularly in the setting of complex MAPCA’s^35,36^.

### Right ventricular outflow tract stenting

#### Background

The modified Blalock-Taussig-Thomas shunt (mBTTS) has largely been superseded by PDA stenting for duct-dependent pulmonary blood flow lesions as it has been shown to be associated with lower morbidity and better pulmonary arterial growth^37–39^. As an extension of this approach, forward PBF may reduce the risk of pulmonary over-circulation or diastolic run-off seen in “shunt physiology” with pulsatile flow optimizing PA growth^40,41^, RVOT stenting in neonates with symptomatic tetralogy has evolved^42,43^. Since its introduction, excellent outcomes, such as low mortality and favorable PA growth, have been reported^42–45^.

#### Indication

RVOT stenting is usually performed in neonates and small infants with symptomatic (increasing cyanosis or hypercyanotic spells) tetralogy of Fallot and its variants. It may be especially beneficial in high-risk groups, namely patients with low birth weight, small or non-confluent PAs, severe genetic disorders, or other extracardiac comorbidities^39^. There has been controversy about the superiority of RVOT stenting to PDA stenting, with the theoretical advantage of forward PBF mentioned above, while drawbacks of RVOT stenting relate to the impact of the stent on definitive surgical repair^35^.

#### Procedure

**Figure 3.**
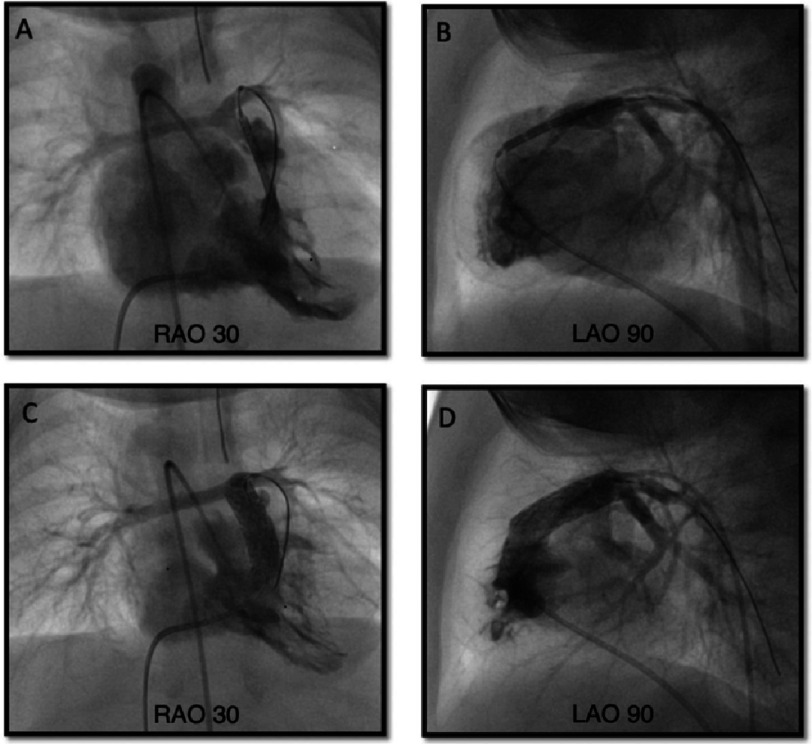
Right ventricular outflow stenting. Upper row: during stent deployment. Lower row: post stent deployment. Abbreviations: LAO, left anterior oblique; RAO, right anterior oblique.

Under general anesthesia, RVOT stenting (Figure 3) is performed from a femoral or jugular venous approach, although the subxiphoid hybrid approach may be considered in patients <2 kgs. Right ventricular angiography, with tube angulation of 30° right anterior oblique with 20° cranial tilt along with a straight lateral is performed to delineate the anatomy. A 0.014” wire is place in the distal branch pulmonary artery and may be “locked” in place with 3–4 clockwise turns. Some centers have shifted away from using longer sheaths because of the potential for hemodynamic instability, particularly in those with hypercyanotic spells. Usually, a stent size of 1–2 mm larger than the diameter of the RVOT in the diastolic phase is appropriate. The stent type may depend on the cath lab’s stocking options. Either a bare metal stent, or a covered stent to mitigate against neo-intimal proliferation, can be used^42,43,45,46^. If a long sheath is not used, angiography can be performed using a 4Fr pigtail catheter retrogradely placed in the RV from the aorta^47^. In smaller infants, transthoracic echocardiography can be used to position the stent. The RVOT should be fully covered with a single long or tandem stent to prevent recurrent progressive muscular obstruction^42^.

#### Postprocedural outcome

There have been a limited number of large studies, and no randomized prospective studies examining the outcomes of RVOT stenting in infants. Currently, two large studies comparing RVOT stenting with surgical procedures from Toronto ( *n* = 42, including 9 TOF with pulmonary atresia)^35^ and Birmingham ( *n* = 60, all TOF)^45^ are available. According to the systematic review which included these two studies and other relatively small case series^48^, the reported successful stent placement rates were 93.6% (95% confidence interval = CI 89.6%–96.2%) and the overall improvement in oxygen saturation was 20.1% (95% CI 15.8%–25.3%). The Birmingham group also illustrated more symmetric branch PA growth in the RVOT stenting group (RPA z-score improved from −2.28 to −0.72, LPA z-score −2.08 to −0.05) compared with the mBTTS group (an increase of PA z-score in the RVOT stenting group was higher as 0.749 for the RPA and 0.599 for the LPA than that of the mBTTS group)^45^.

Procedural complications such as stent migration, tricuspid injury, bradyarrhythmias, and aortic deformation have been reported^44,46,49,50^. Although coronary abnormalities exist in 5–7% of TOF patients^1^, previous reports identified up to 10 patients with an abnormal coronary course; however, none of these demonstrated coronary ischemia related to RVOT stenting^51^. Mortality rates after RVOT stenting have been reported to be 2–5%^35,44^, similar to those after mBTTS^48^. According to the Toronto report, mortality after RVOT stenting (2/42) was similar to that after early (<3 months) surgical repair and elective (3-11 months) primary surgical repair (1/45). It should be noted that these studies may have encompassed selection bias, namely, the patients in the RVOT stenting groups in these studies had more severe status such as smaller PAs, multiple comorbidities^35^, prematurity, small-for-gestational-age, or gut/brain abnormalities^44^ than other groups. No direct comparative studies have been conducted on RVOT stenting and surgical repair in high-risk patients.

### Bridging to the repair therapy

Re-intervention rates after RVOT stenting have been reported to be 41–56%, higher than those after mBTTS palliation^44^ or after surgical repair performed between 3-11 months (no difference between the RVOT stenting group and the early surgical groups)^35^. However, a further study from Toronto demonstrated that the risk of surgical reoperation was associated with early surgical repair, and not with the early catheter RVOT palliation^52^. Most of the reinterventions were transcatheter in nature due to RVOT obstruction (balloon dilation or repeat stent placement)^35,44,50^. Bare metal stent-induced tissue reaction and fibrous tissue ingrowth may have contributed to this obstruction. It is unclear whether drug-eluting stents or bioresorbable stents mitigate this reaction^44,53^.

The technical burden of definitive surgical repair following RVOT stenting is another concern. RVOT stents may require a transannular patch (TAP) repair procedure and/or relatively broad RVOT resection to retrieve the stent. In the Toronto report, none of the patients after RVOT stenting underwent valve-sparing repair^35^ whereas 41% of the early repair (TOF with pulmonary stenosis) group achieved valve-sparing surgery. However, there were differences in baseline pulmonary valvular size between the two groups (median z-score of −6.6 in the former group *vs* −4.4 in the latter group). Furthermore, there was no difference in the rates of valve-sparing surgery or pulmonary valvular size between the RVOT stenting group and the early repair for TOF with pulmonary atresia group. A similar result was shown in the Birmingham report, as a valve-sparing surgery rate of 7% after RVOT stenting was not significantly different from that of post-mBTTS patients (10%)^44^. Indeed, the Birmingham group moved to subvalvular stent placement to avoid the pulmonary valve; however, the rate of transannular patch placement remained high. Stent removal at the time of surgery may be influenced by the length of time the stent remains in place. Complete retrieval of the RVOT stent was reported in 95% of patients approximately 6 months after initial stent implantation, whereas in another report, only 47% of patients underwent complete retrieval at a median of 220 days following implantation^53^. It is unclear whether covered stent implantation reduces intimal ingrowth and ameliorates stent removal at the time of surgical repair.

## Summary

Although RVOT stenting is a novel technique predominantly performed in high-risk patients, its safety and efficacy have been demonstrated. However, high reintervention rates and detrimental effects on the final surgical repair remain unresolved problems.

## Transcatheter pulmonary valve replacement (TPVR)

### Historical overview

After surgical repair of RVOT abnormalities, typically represented by TAP RVOT reconstruction for TOF or RV to PA conduit implantation for pulmonary atresia, most patients have RV remodelling secondary to pulmonary regurgitation (PR) or multi-level RVOT obstruction. These lesions are usually progressive, and consequent RV dilation or dysfunction is irreversible and leads to adverse clinical outcomes, such as heart failure, refractory tachyarrhythmias, or sudden cardiac death (SCD)^54–56^. Therefore, prophylactic repair of these lesions, such as pulmonary valve replacement, is essential. However, even a successful surgical valve replacement may not be definitive, and patients usually require several lifetime surgical valve replacements. Moreover, an increased number of open-heart surgeries are associated with morbidity and mortality^57–59^.

In 2000, Bonhoeffer et al. reported the first case of TPVR using a bovine internal jugular vein with a native valve mounted on a platinum iridium stent^60^, with the following case series demonstrating excellent procedural outcomes^61,62^. This technique led to the establishment of the Melody Transcatheter Pulmonary Valve (Medtronic Inc., Minneapolis, MN, USA)^63^, and the device received the European CE mark in 2006 and Food and Drug Administration (FDA) approval in the United States in 2010. Since then, with 20 years of cumulative experience, technical advances, and the introduction or invention of other novel valves, TPVR is currently a widely accepted, less invasive alternative repair treatment to SPVR, reducing the number of open-heart surgeries required during the patient’s lifetime.

### Preprocedural evaluation

Currently, TPVR is performed using indications similar to those used in SPVR. For patients who have repaired CHD, such as TOF with cardiovascular symptoms attributable to significant PR^64–66^ or RVOT obstruction^64,65^, most clinical guidelines recommend pulmonary valve replacement (PVR). The European Society of Cardiology (ESC) guidelines specifically recommend performing TPVR in patients with non-native RVOT morphology^65^. However, the treatment indications for asymptomatic patients with significant PR or RVOT obstruction are controversial. Valve replacement is considered in cases of severe RV enlargement with a significantly elevated right ventricular end-diastolic volume (RVEDV) index to body surface area, right ventricular end-systolic volume (RVESV), or dysfunction of the RV on cardiac magnetic resonance imaging (CMR) (see Table 2).

**Table 2:** Recommendations for pulmonary valve replacement for repaired tetralogy of Fallot.

	**ACC / AHA 2018**	**ESC 2020**	**CCS 2022**
**Recommendation**	**Indication for pulmonary valve replacement**		
**Class I, or strong in CCS** **= symptomatic patients**	≥moderate PR	severe PR^*^ and/or ≥ moderate RVOTO^a^ TPVR is preferable than SPVR in ”non-native” RVOT patients	≥ severe PR^b^
**Class IIa, or weak in CCS** **= asymptomatic patients**	≥moderate PR with any 2 of RV enlargement ≥ mild LV or RV dysfunction stenosis RVSP ≥ 2/3 systemic objective exercise intolerance	severe PR and/or RVOTO with any of progressive RV enlargement RVOTO: RVSP > 80 mmHg progressive RV dysfunction objective exercise intolerance	severe PR with RV enlargement severe PR with any of concomitant lesion lesion require intervention sustained VT
**Description**			
**RV enlargement**	RVEDV ≥ 160 ml/m^2^ and/or RVESV ≥ 80 ml/m^2^ and/or RVEDV ≥ 2 x LVEDV	RVEDV ≥ 160 ml/m^2^ and/or RVESV ≥ 80 ml/m^2^ and/or progression of TR ≥ moderate	RVEDV ≥ 160 ml/m^2^ and/or RVESV ≥ 80 ml/m^2^

**Notes.**

Abbreviations ACC American College of Cardiology AHA American Heart Association CCS Canadian Cardiovascular Society CMR cardiac magnetic resonance imaging ESC European Society of Cardiology LV left ventricle LVEDV left ventricular end diastolic volume PR pulmonary regurgitation RV right ventricle RVEDV right ventricular end diastolic volume RVESV right ventricular end systolic volume RVOTO right ventricular outflow tract obstruction RVSP right ventricular systolic pressure SPVR surgical pulmonary valve replacement TPVR transcatheter pulmonary valve replacement TR tricuspid regurgitation VT ventricular tachycardia

* Regurgitant fraction by CMR > 30–40%.

a Peak velocity > 3 m/s.

b Severe PR on echocardiography or a regurgitation fraction > 25% on CMR.

Initially, TPVR was indicated for patients aged ≥5 years and weighing ≥30 kg^67^ because of the large-calibre delivery system *via* the femoral vein; nevertheless, TPVR *via* femoral access is well tolerated for patients weighing <30 kg^68^. TPVR can be performed for a very small patient (*e.g.*, <20 kg) with limited venous access, such as venous obstruction, *via* the transjugular, transsubclavian^68,69^, or transhepatic venous approach^70^, technical challenges remain, and hybrid pulmonary valve replacement can be more straightforward for a very small patient, particularly with complex anatomy of the RVOT^71–73^ or in a bailout situation^74–76^.

Understanding the anatomy of the valve landing zone with surgical history taking and multimodality imaging such as computed tomography (CT) or CMR is essential^77,78^. The shape of the RVOT, smallest diameter of the RVOT in systole, narrowest and largest diameter of the conduit, length of the RVOT or conduit, distance to PA bifurcation, anatomy and proximity of the CA, distance from the landing zone to the sternum, and calcification of the conduit or RVOT tissue should be assessed^77,79^.

Based on this information, a consensus from a multidisciplinary team, including interventionists, surgeons, ACHD specialists, and anesthetists, should be obtained before the procedure.

### Preparation: RVOT rehabilitation, Valve in Valve, and native RVOT

Prior to the valve deployment, a full hemodynamic study, angiography in the RVOT, dilation of a compliant sizing balloon in the valve landing zone and simultaneous coronary angiography or aortography to rule out CA and aortic compression should be carried out^64,65,80–83^. Three-dimensional rotational angiography (3DRA) is useful for better visualization of RVOT morphology, CA or aortic compression during the balloon dilation test. Despite concerns about increased radiation exposure with 3DRA, it has been reported that the total radiation dose is no different to conventional bi-plane angiography^84,85^.

If RVOT obstruction exists prior to the valve implantation, RVOT rehabilitation and elimination of the obstructive lesion with a stent are essential. Elevated RV pressure has been reported to be an independent risk factor for death^86,87^ and ventricular tachyarrhythmia^88^. Furthermore, recent long-term follow-up studies of TPVR illustrate that the pressure gradient across the RVOT is a key risk factor for the development of valvular dysfunction or infective endocarditis (IE)^89,90^. Pre-stenting of the conduit prior to valve deployment is a common procedure for the Melody valve, as it helps provide a suitable landing zone and can reinforce radial strength to prevent frame fracture of the Melody valve^91–95^. To reduce prestent-related technical difficulties or risk of stent embolisation or displacement^96^, simultaneous stenting techniques, crimping of one or more bare metal stents over the valve, and deployment of the whole system in one step have been introduced^97–99^. Conduit injury is reported in approximately 5% of cases and can occur during RVOT rehabilitation, such as pre-stenting or TPVR, necessitating covered stent implantation^100–102^.

Valve-in-valve replacement in a surgically implanted bioprosthetic pulmonary valve (BPV) is effective and generally reported with less CA compression or frame fracture^103–105^, because the rigid frame of the BPV protects the new valve inside^92,106^. It is imperative to understand the accurate type and size of the previously implanted BPV, as well as the nominal diameter, which typically represents the outer diameter. The inner diameter varies depending on the BPV type^107^. If the inner diameter is insufficient to the estimated adequate pulmonary valvular size^108^, intentional frame fracture of the BPV can be performed with a high-pressure balloon up to the outer diameter or 1-2 mm larger than the BPV^106^. In such situations, consideration of CA compression is crucial.

Approximately three-quarters of patients with repaired CHD and RVOT abnormalities have a “native” outflow, which includes a patched RVOT anatomy. However, because the native RVOT usually has a dilated, complex, and heterogeneous morphology^78,109^ caused by the surgical scar and subsequent dyskinesis or aneurysmal changes in the RVOT^110^, it is not always straightforward to perform TPVR for native RVOT. There are five main types of RVOT anatomy: pyramidal, straight, funnel, convex, and hourglass shape^78^. The most common pyramidal shape has a dilated inflow, which is also the most difficult anatomy because of the lack of an adequate landing zone for the valve^79^. Technical attempts have been made, such as pre-stenting or surgical plication of the RVOT prior to the Melody valve and SAPIEN THV deployment^110,111^. To meet this demand, self-expanding alternatives such as the Venus P valve, Harmony valve, and Alterra Prestent have been introduced in the past decade.

### Valve deployment

**Figure 4.**
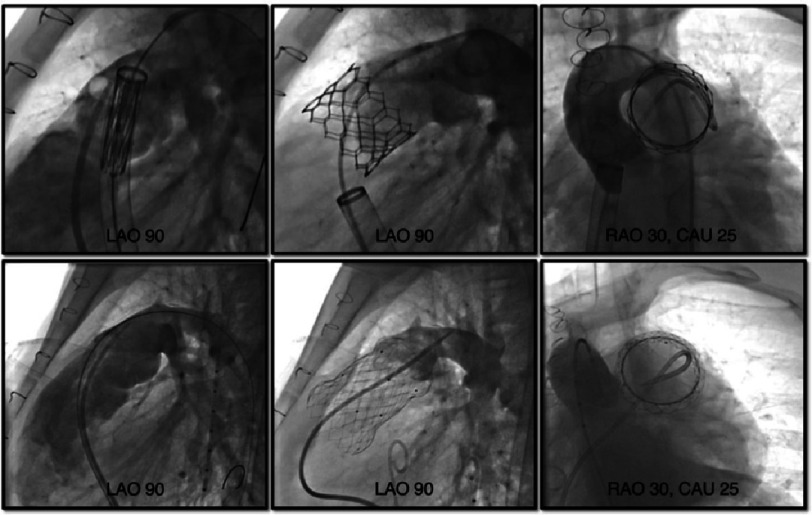
Transcatheter pulmonary valve implantation. Upper row: for SAPIEN S3 valve, Lower row: for Venus P valve. From left to right: Valve deployment, pulmonary angiography post deployment, aortic angiography post deployment. Abbreviations: CAU, caudal angulation; LAO, left anterior oblique; RAO, right anterior oblique.

The valve system is delivered to the target area over a stiff guidewire in the distal PA, usually left PA is preferable with better trajectory and tracking (Figure 4). With stiffer delivery systems or dilated and complex anatomy of the RVOT and PA bifurcation, delivery of the valve can be challenging^112^. In such situations, one may consider the anchor balloon and buddy wire technique^113^ or utilize a kink-resistant, flexible long sheath such as GORE Dryseal (W. L. Gore & Associates, Inc, Delaware, USA) sheath^112,114,115^. The device preparation and deployment varies depending on the device (next section), and post-implant hemodynamic data and pulmonary angiograms are obtained.

### Balloon-expandable valve

The Melody valve (Medtronic Inc) is the first transcatheter implantable valve which consists of a trileaflet bovine jugular venous valve sutured inside a bare-metal Cheatham-platinum (CP) stent (NuMED, Inc., Hopkinton, New York) with gold-blazed welds. Currently, two valve sizes, 16 and 18 mm in diameter, are available, and the valve is delivered *via* the 22 Fr Medtronic Ensemble delivery system, which utilizes a balloon-in-balloon (NuMED, Inc., Hopkinton, New York) with 18, 20, and 22 mm outer balloons. Each valve size can be expanded and deployed up to 20 mm and 22 mm respectively^63^. The procedural success rate is 90–98% with excellent acute or mid-term results, achieving reduction of RVOT obstruction and excellent relief of PR^89^. Frame fracture, which leads to valve dysfunction or recurrent RVOT obstruction, is one of the biggest concerns; however, with the establishment of pre-stenting techniques^91–94^, the incidence of stent fracture has significantly decreased^93^.

SAPIEN XT and SAPIEN 3 (Edwards Lifesciences LLC, Irvine, California, USA) transcatheter heart valves (THVs) are other balloon-expandable valves which received CE marks in 2016 and 2021, respectively, and approval of the US FDA in 2016 and 2020, respectively. These THVs comprise integrated, unidirectional, trileaflet bovine pericardial tissue valves mounted within cobalt chromium stents and polyethylene terephthalate (PET) fabric cuffs.

The SAPIEN XT THV has three sizes of 23, 26, and 29 mm, and the SAPIEN 3 THV has an additional size of 20 mm. This wide range of sizes allows for implantation of SAPIEN THVs for various landing zone diameters, including native or patched RVOT’s^116,117^. The SAPIEN 3 THV also has a specific refined design of externally sealed PET skirts which may diminish paravalvular leaks and longer valve length for better stabilization^118^. The valve is crimped with an Edwards crimper and delivered *via* the Edwards Novaflex delivery system for SAPIEN XT or Edwards Commander Delivery system for SAPIEN 3. The characteristics of the three expandable valves are summarized in Table 3.

**Table 3:** Currently available balloon-expandable valves.

	**Melody**		**SAPIEN XT**			**SAPIEN 3**			
Appearance	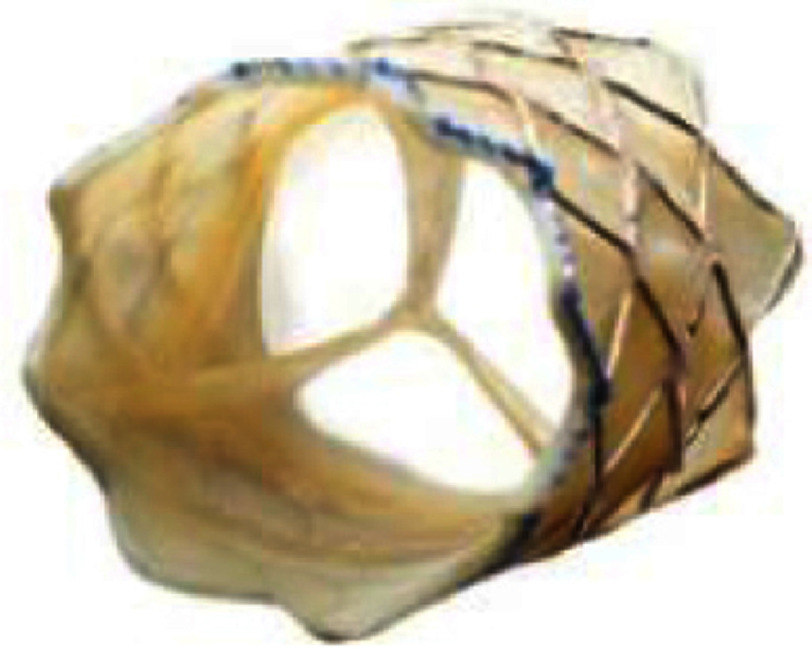		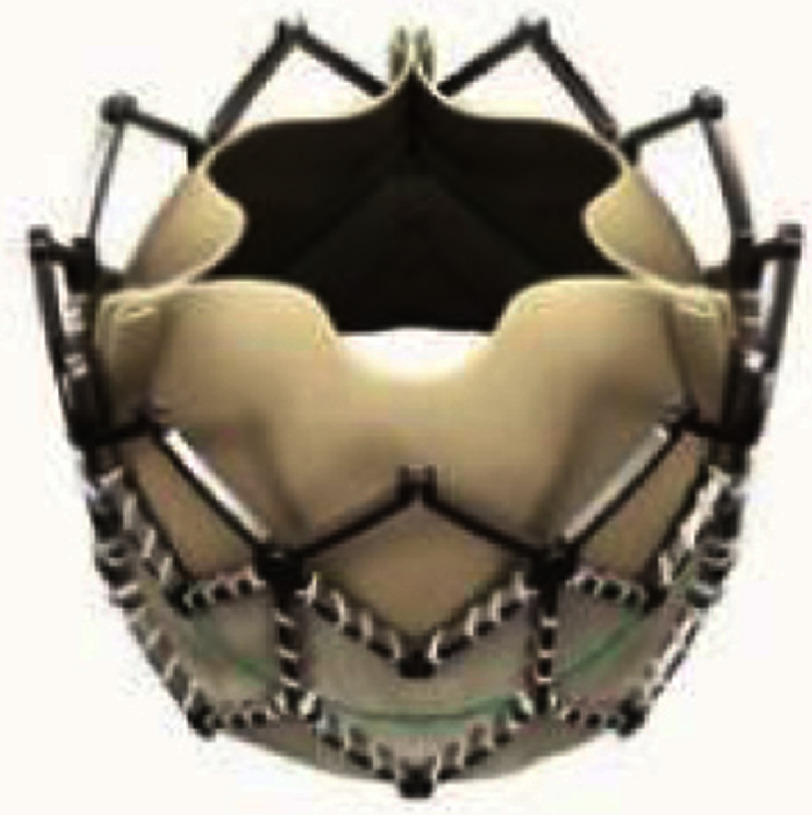			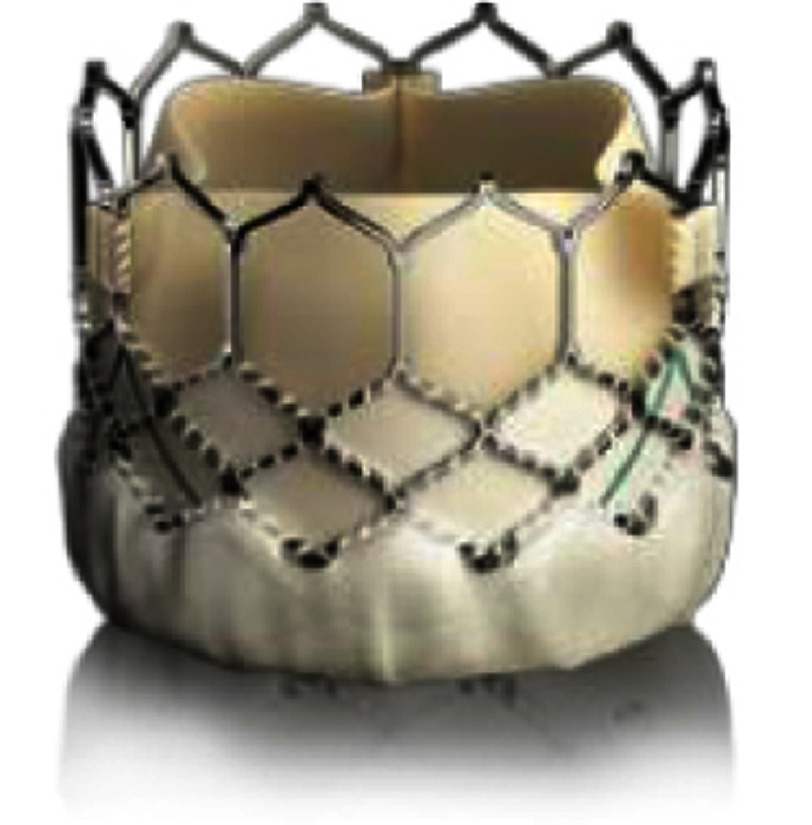			
Valve size, mm (balloon size^*^)	16 (18-20)	18 (18-22)	23	26	29	20	23	26	29
Valve height: unexpanded, mm	28	30	14.3	17.3	19.1	15.5	18	20	22.5
Appropriate RVOT diameter, mm	≤20	≤22	18-22	21-25	24-27	16-19	18-22	21-25	24-28
Delivery sheath size, Fr	22		16, 18, 20			14, 16 (for 29 mm S3)			
Suitable landing zone anatomy	conduit (with prestent), bioprosthetic valve		conduit, native RVOT, bioprosthetic valve for stenotic lesion longer than the valve, place prestent						

**Notes.**

Abbreviations RVOT right ventricular outflow tract

* Only different for the Melody valves.

The COMPASSION and COMPASSION S3 trials were prospective multicenter studies that demonstrated the safety and efficacy of SAPIEN XT THV and S3 THV prior to FDA approval. Implantation data from 69 patients and 3-year follow-up data from 57 patients were evaluated in the COMPASSION study. At 3 years, freedom from all-cause mortality, reintervention, and endocarditis were 98.4%, 93.7%, and 97.1%, respectively. The mean peak conduit gradient decreased from 37.5 mmHg to 17.8 mmHg and PR was mild or less in 91.1% of patients. Functional improvement in New York Heart Association (NYHA) functional class was observed in 93.5% of patients^100^.

In the COMPASSION S3 trial, 56 patients underwent implantation and 51 patients were followed up by one-year. Valve implantation was successful in 98.2% of the cases on the first attempt, and only one case required a second S3 THV. There was no immediate residual obstruction, defined as a peak gradient across the RVOT of ≥35 mmHg or ≥ moderate PR. One year follow-up results were excellent, with no mortality, IE, valve explant or reintervention, thrombosis, or frame fracture. The THV function on echo were favorable at one-year, mean RVOT gradient decreased from 28.0 mmHg to 14.2 mmHg and only one patient (2.1%) had a mean gradient ≥ 40 mmHg. 97.9% of patients had mild or less PR. Paravalvular leak was noted in 6.4%. Functional improvement in NYHA class was also reported and all patients remained less than class II (90.2% with class I and 9.8% with class II) at one year follow up^119^.

A multicenter retrospective study of 774 patients, which included 397 (51%) of patients with a native RVOT, demonstrated successful implantation in 97.4% patients and serious adverse events, including death in 2 cases, stent embolization or malposition, coronary or arterial compression, or tricuspid valve injury, in 67 (10%) patients, regardless of RVOT morphology^116^. The low profile of the Edwards Expandable Sheath (eSheath) set a minimum 14 Fr requirement, however it necessitates a slightly complicated maneuver of *in vivo* balloon loading to the eSheath and valve delivery without coverage^119^, which consequently may cause tricuspid valve injury. The prevalence of this has been reported as high as 6−7.5% during the valve passage^118,120,121^. Use of the Dryseal long sheath can decrease this complication^115,122^, and has become a standard technique in most centers. No frame fractures have been reported in the pulmonic position; hence, pre-stenting is not always necessary for SAPIEN THVs^119,121^. Subclinical leaflet thrombosis on CT is associated with increased gradients and adverse clinical events following transcatheter aortic valve replacement. This hypoattenuating leaflet thickening has been reported in post-TPVR patients with early valve failure^123,124^.

### Self-expanding valves and a prestent system

The most important advantage of a self-expanding valve is the availability of large sizes to fit the dilated native RVOT. However, in the presence of concomitant RVOT stenosis, the use of a self-expanding valve may not provide a sufficient radial force. To ensure valve stability and reduce paravalvular leak, the deployed valve size is usually slightly larger in diameter than the narrowest diameter of the RVOT landing zone. The Harmony (Medtronic Inc.) transcatheter pulmonary valve (TPV) is a self-expanding valve for larger native RVOT’s, approved by the US FDA in 2021 after clinical trials under the FDA Early Feasibility Study (EFS) pathway and two subsequent studies, the Harmony pivotal study and Continued Access Study (CAS)^125,126^.

The valve has a 22 (TPV 22) or 25 (TPV 25) mm porcine pericardium valve with anti-mineralization treatment to minimize calcification which is sewn into a nickel-titanium (nitinol) frame covered with a polyethylene cloth. Both TPVs have asymmetrical hourglass shapes with larger proximal flaring to hold the enlarged RVOT. TPV 25 had a slightly shorter length of 51 mm, in comparison to 55 mm of TPV 22 and a wider base skirt of 54 mm (Table 4). Both TPVs are delivered *via* a 25 Fr coil loading delivery system which has good trackability. When delivered to the target area of the RVOT and unsheathed, the valve expands from its distal end. The valve can be released by rotating the handle of the delivery system. Fit analysis to evaluate the adequacy of Harmony valve placement is mandatory and is performed by utilizing ECG-gated CT angiography images with reconstruction in both systole and diastole. Early term results of the pivotal study and the CAS of 87 patients described favorable outcomes without procedure- or device-related death and reintervention-free survival in 98% of TPV 22 patients and in 91% of TPV 25 patients within one year^127^. The longevity of the valve is unclear; however, a 5-year follow-up data of 20 patients in EFS reported reintervention-free rates of 75% with two valves in valve TPVR’s, two surgical valve replacements, and one mid-term death^128^. Ventricular tachycardia (VT) was reported in 16% to 40% of patients with non-sustained VT and in 3% of patients with sustained VT, which may be related to device extension below the annular valve position^127,129^.

**Table 4:** Currently available (approved or investigational) self-expanding valves and the self-expanding prestent for native right ventricular outflow tract.

	**Harmony TPV**		**Venus P-valve**	**Pulsta valve**	**PT valve**	**Alterra stent**
Appearance (outlet flair = upper in the right images)	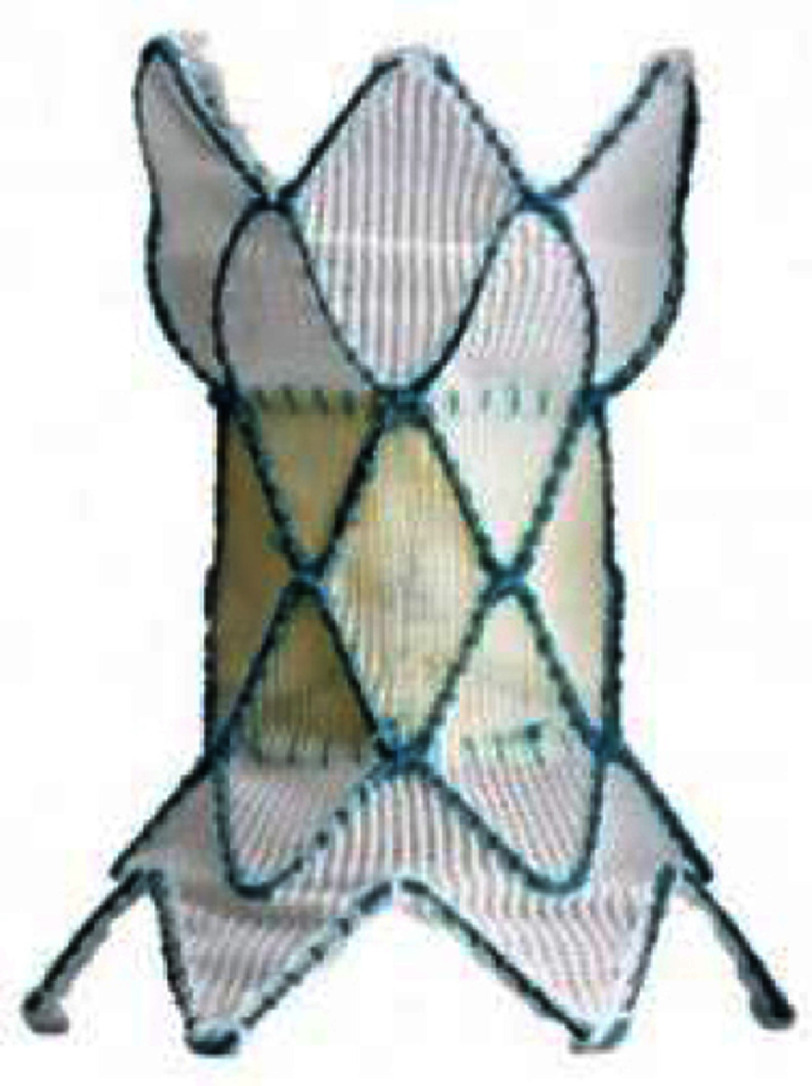	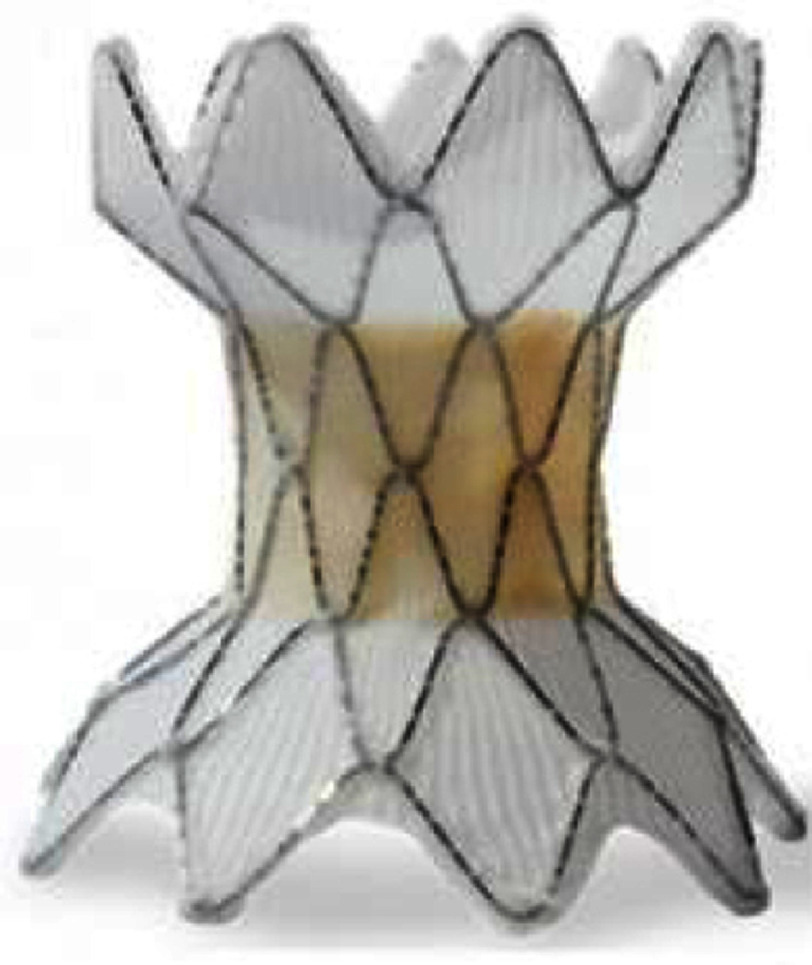	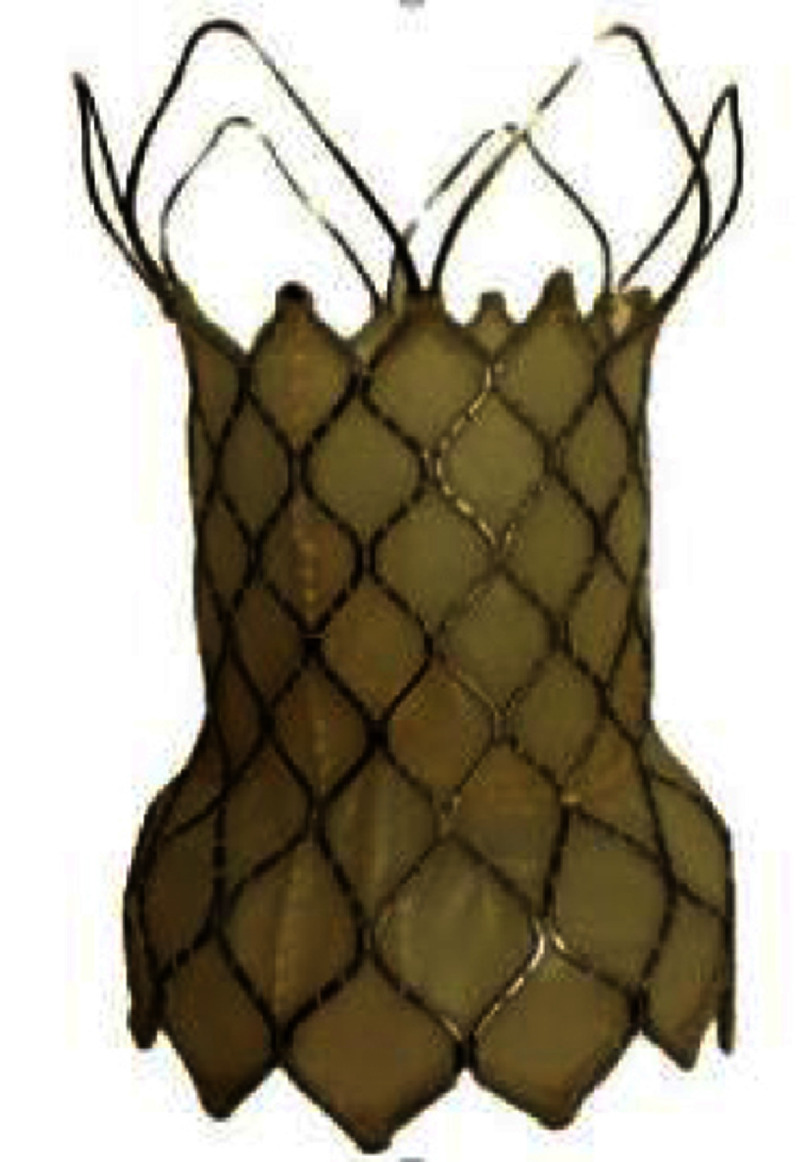	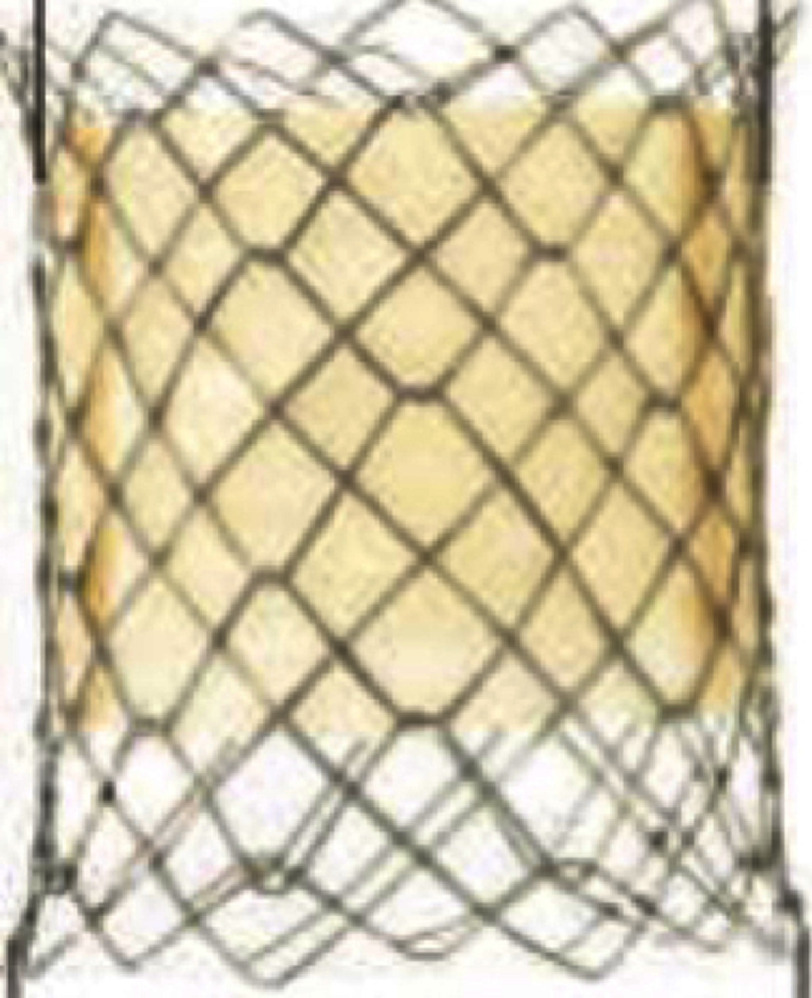	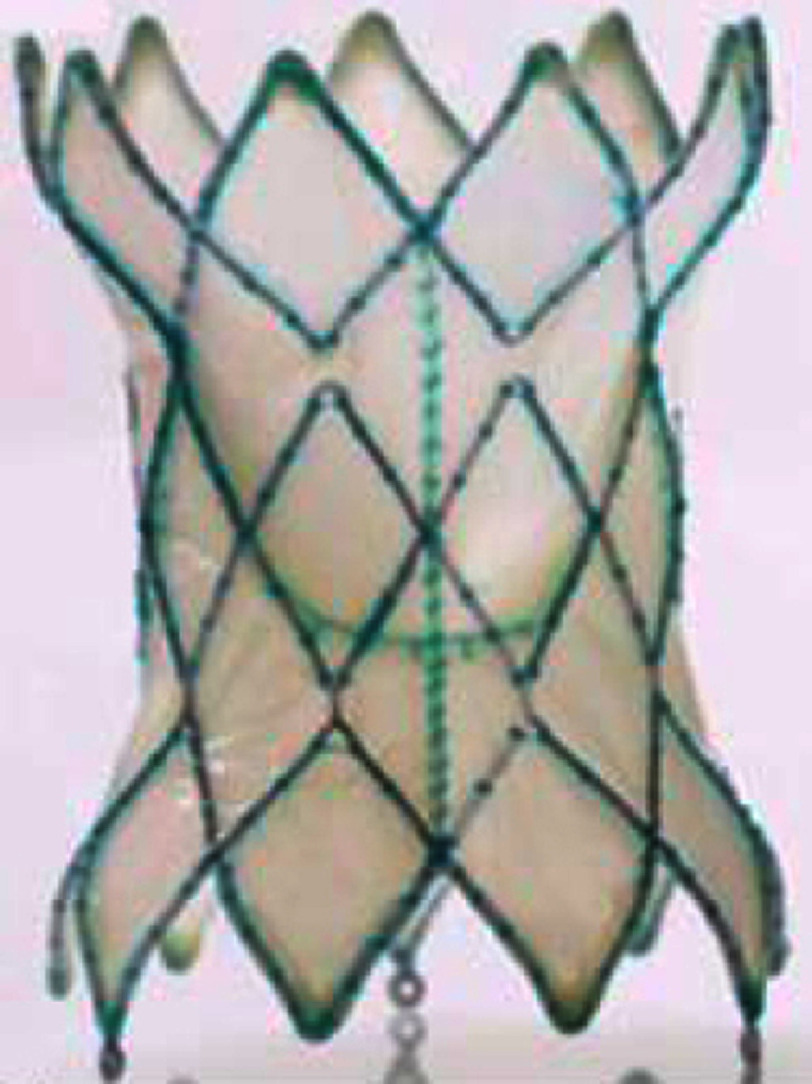	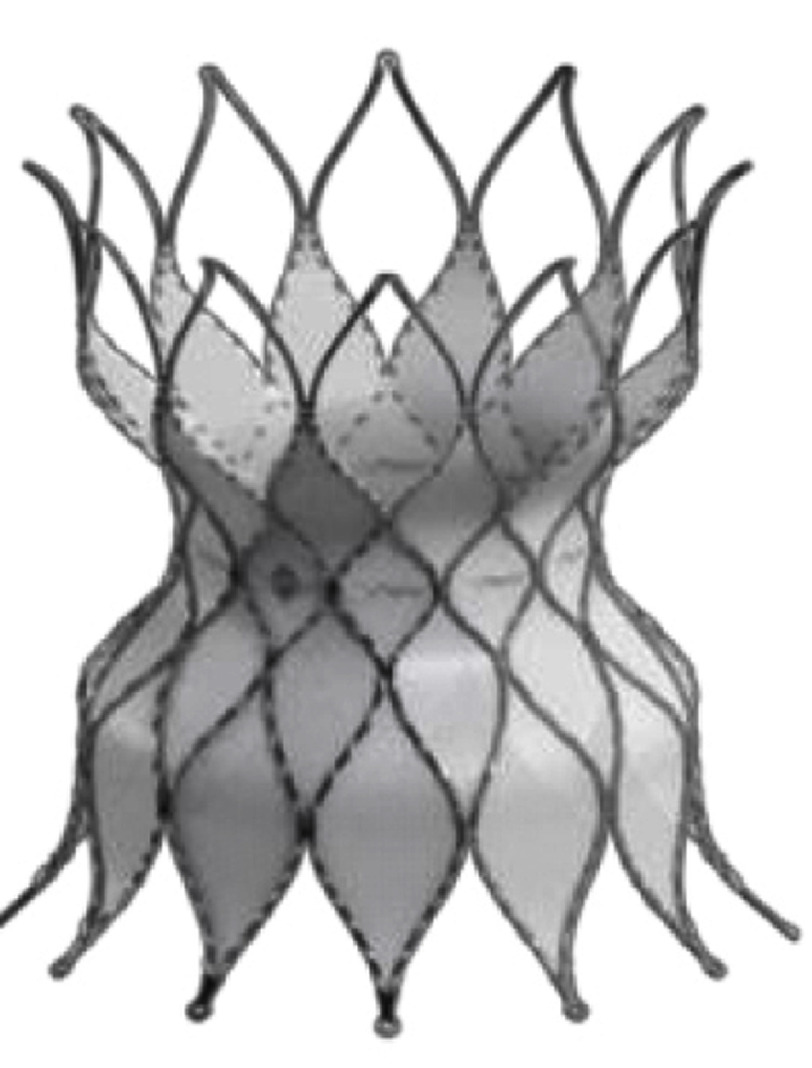
Valve size, mm	22	25	16-36 (each 2)	18-28 (each 2)	20, 23, 26	SAPIEN S3 29
Middle waist , mm	= valve size	= valve size	28-46 (each 2)	22-32 (each 2)	38-54	27
Inlet flair, mm	41	54	+10 mm of waist	+4 mm of waist	28-44 (each 4)	40 mm
Outlet flair, mm	32	43	same as inlet	same as inlet	same as inlet	same as inlet
Total length mm	55	51	25, 30^*^	28, 31, 33, 38	38-54	49^a^
Coverage	full covered	full covered	proximal only	waist only	full covered	proximal only
Delivery system	25 Fr	25 Fr	22, 24 Fr	18, 20 Fr	21 Fr	16 Fr
Recapture	26 Fr DS	26 Fr DS	unknown	own system^b^	unknown	own system^c^

**Notes.**

Abbreviations TPV transcatheter pulmonary valve DS Gore® Dry seal sheath

* Proximal covered length.

a Proximal covered length = 30 mm.

b Up to 30% of deployment.

c Up to 50% of deployment.

The Venus P-valve (Venus MedTech, Hangzhou, China) is a self-expanding valve which received European CE mark in 2022. The valve is comprised of a porcine pericardium valve with proximal and distal flare-shaped nitinol self-expanding covered stents to fit the native RVOT. Only the distal flare portion is uncovered to facilitate blood flow into the branch pulmonary arteries. Currently available sizes range from 28 to 36 mm diameters in two mm increments, sized 2 to four mm larger than the balloon dilated RVOT diameter. Each valve diameter size has two available stent lengths of 25 and 30 mm. The delivery system is 22 or 24 Fr.

Previous reports have demonstrated challenges with delivery with previously placed left PA stents or sharply angled RVOT’s^130^^131^; however, utilizing the Dryseal sheath might provide more predictable delivery of the valve in such situations^112^. The early- and mid-term results of the Venus P-valve are excellent, with a 91–98% success rate with high freedom from valve dysfunction between 2–5 postprocedural years despite a 15–26% incidence of stent frame fractures^130–132^.

The Alterra Adaptive Prestent (Edwards Lifesciences) was designed for use as a docking adaptor for the 29 mm SAPIEN 3 THV within the RVOT. It comprises a self-expanding nitinol frame of symmetrically shaped inflow and outflow flaring with a PET fabric covering. Similar to the Venus-P valve, the distal outflow stent is uncovered to avoid peripheral PA obstruction. The central section is 27 mm in diameter, and both flared portions are 45 mm in diameter to provide firm holding to the RVOT. Although the total unconstrained device length is 48 mm, the completely covered length is only 30 mm which can be applied to shorter native RVOT’s. The optimal landing zone size is between 27 mm and 38 mm in diameter and ≥ 35 mm in length. The delivery system is designed to provide slow, controlled deployment and recapture within two attempts by utilizing a one-handed rotational knob^133^. An early feasibility study of this system demonstrated 100% successful deployment in 15 patients, with bleeding in four patients and one event of atrial fibrillation^134^.

### Upcoming valves

Although currently not approved in either the U.S. or Europe, various novel valves are being evaluated in other countries. The Pulsta valve (TaeWoong Medical Co., South Korea) is a self-expanding valve system. Its unique feature is absence of the hourglass shape like other self-expanding systems. The valve comprises a porcine pericardium valve sutured on a knitted double-stranded Nitinol wire frame. Both the proximal and distal ends are uncovered and mildly flared four mm wider than the body of the valve, which is designed to avoid peripheral PA obstruction and to provide stable positioning. The valve is available in sizes of 18–28 mm with two mm increments. The length of the stent is 28–38 mm. The valve is crimped onto the 12 Fr delivery cable using the Heart Valve Crimper (Model RVS, Blockwise Engineering LLC). During mounting, the proximal frame is hooked to a “hook block” at the proximal part of the valve-loading area to provide controlled release and prevent abrupt valve jumping. The outer size of delivery sheath is 18-20 Fr^135^. An early- and mid-term report of Pulsta valve in 25 patients with significant PR described 100% procedural success and no mid-term valve stenosis or significant PR. The mean RVEDV index decreased from a mean of 169.7 to 126.9 ml/m^2^ after 6 months^136^.

The Med-Zenith PT valve (Beijing Med-Zenith, China) is also a self-expanding valve with a porcine pericardial valve and a symmetrically shaped nitinol wire frame, and the valve is entirely covered with porcine pericardium. This is made for native RVOT and the clinical trial is still ongoing in China. Similar to other self-expanding systems, a preprocedural fit analysis is required. Twenty-two of the 37 patients who met the fit analysis criteria underwent implantation with the PT valve in a Chinese clinical trial. Valve implantation was successful in all patients without acute device embolization or malposition, coronary compression, ventricular arrhythmias, or tricuspid valve injury. RVEDV index decreased from mean of 181.6 to 123.4 ml/m^2^ after 1 year^137^.

### Complication

Coronary artery compression, which usually involves the left main/left anterior descending branches, can be catastrophic and occurs in approximately 5% of patients during the balloon dilatation test^138,139^. Abnormal CA anatomy, altered CA position due to previous CA reimplantation or displacement of the aorta, or close distance between the CA and the landing zone of the valve are known substantial risks for this complication^81,140^. Therefore, it is important to delineate the origins and proximal courses of the coronary arteries before the procedure^64^; however, expected CA compressions cannot be reliably simulated, and balloon assessment is still essential. As mentioned previously, 3DRA is also helpful^84^. Aortic root compression is another important concern, and this complication has been documented^83^. This complication is more frequent with TPVR for native RVOT’s than for conduits, and significant deformation of the aortic root with aortic regurgitation is a contraindication for TPVR^110,118^.

Infective endocarditis is a widely recognized late complication that affects valve function and longevity. The annualized incidence of post-TPVR infective endocarditis with the Melody valve has been reported to be approximately 1.6–4%^141–144^ and the largest retrospective study to date included 2,476 patients and showed an IE incidence of 16.9% after TPVR after a median follow-up of 8 years^145^. However, there were no significant differences in the estimated survival free of endocarditis or survival free of valve replacement due to IE between the Melody TPVR group ( *n* = 241) and SPVR group ( *n* = 211) in a comparative study^141^. It is important to realize that IE after TPVR may be treatable with IV antimicrobial therapy, and explants of the valve may only be required if there is significant valve dysfunction^146^. Various risk factors of IE have been advocated, with another multicenter retrospective study of 845 post Melody TPVR patients revealing invasive RV-PA pressure gradients (per five mmHg) as an independent risk factor of IE (adjusted hazard ratio HR: 1.19, 95% CI 1.07–1.32; *p* =*0.002*)^89^. Despite the difficulty of direct comparison because of the different backgrounds of patients or institutions, several reports have suggested a low incidence of IE with SAPIEN THVs when compared with the Melody valve^100,118,119,122^, although this may be related to different subtypes of RVOT dysfunction.

### Long term outcome

Currently, long-term outcomes are mostly available for the Melody valve and the SAPIEN valve. Jones et al. ^90^ reported 10-years follow up experience of 150 patients with the Melody valve, with a freedom from mortality rate of 90%, and freedom from any reintervention of 60%. They described age ≤21 years at time of implant (HR: 2.2, 95% CI 1.1–4.3; *p* =0.03), a primary indication of stenosis (HR: 2.7, 95% CI 1.3–5.4; *p* =0.006), the number of prior open-heart surgeries (HR: 1.3, 95% CI 1.0–1.8; *p* =0.06), higher post-implant RV-pulmonary artery peak-to-peak pressure gradient (HR: 1.1, 95% CI 1.0–1.1; *p* =0.006) as independent risk factors of reintervention. McElhinney et al. ^147^ also illustrated 8 years results looking at reintervention and survival after TPVR with both the Melody valve and the SAPIEN valve. There were 95/2476 (3.8%) deaths with 25% related to heart failure, 13% IE, and 7% related to procedural complications, respectively. The risk factors for death were age at TPVR (HR: 1.04 per year, 95% CI 1.03–1.06 per year; *p*¡0.001), a prosthetic valve in other positions (HR: 2.1, 95% CI 1.2–3.7; p 1/4 0.014), and an existing transvenous pacemaker/implantable cardioverter-defibrillator (HR: 2.1, 95% CI 1.3–3.4; P 1/4 0.004). In their cohort 258/2476 reinterventions were performed and risk factors for reintervention were age at TPVR age (HR: 0.95 per year, 95% CI 0.93–0.97 per year; *p*¡0.001), prior endocarditis (HR: 2.5, 95% CI 1.4–4.3; p 1/4 0.001), TPVR into a stented bioprosthetic valve (HR: 1.7, 95% CI 1.2–2.5; P 1/4 0.007), and post-implant gradient (HR: 1.4 per 10 mmHg, 95% CI 1.2–1.7 per 10 mmHg; *p* < 0.001). These reports suggested that TPVR is an effective alternative to SPVR with acceptable longevity.

### Summary

Following the gground-breaking changes introduced by Bonhoeffer, TPVR has become one of the most important therapies for patients with congenital RVOT abnormalities. Consequently, it is now possible to provide complete percutaneous “repair” of complex RVOT abnormalities. Future advances in valve technology, including the potential for fetal valve replacement with bioengineered valves, may overcome issues surrounding somatic growth and valve degeneration. It is conceivable that percutaneous repair of the subtypes of tetralogy of Fallot may be feasible within a generation. Meanwhile, continued collaboration with our surgical colleagues to find less invasive and effective solutions for our patients is key.
